# Anti-Lung Cancer Activity of the Curcumin Analog JZ534 In Vitro

**DOI:** 10.1155/2015/504529

**Published:** 2015-04-21

**Authors:** Jianzhang Wu, Zhijian Cai, Xiaoyan Wei, Minxiao Chen, Shilong Ying, Lingyi Shi, Ren-Ai Xu, Fan He, Guang Liang, Xiuhua Zhang

**Affiliations:** ^1^Chemical Biology Research Center, School of Pharmaceutical Sciences, Wenzhou Medical University, Wenzhou, Zhejiang 325035, China; ^2^Department of Pharmacy, The First Affiliated Hospital of Wenzhou Medical University, Wenzhou, Zhejiang 325000, China

## Abstract

This study investigated the anticancer effect of the curcumin analog JZ534 on lung cancer cell lines H460, A549, H1975, and HCC827. The antiproliferation effect of JZ534 was measured through the methylthiazoletetrazolium assay, and cell colony formation was observed. Cell cycle and apoptosis were determined by flow cytometry, and the preliminary mechanism was studied by Western blot. Results showed that JZ534 significantly inhibited the vitality and colony formation of lung cancer cells. JZ534 induced the G_2_/M cell cycle arrest of the cancer cells and suppressed the expression of cycle-related proteins, including cyclin B1 and Cdc2. Meanwhile, JZ534 induced cell apoptosis and upregulated the expression of apoptosis-related proteins, including cleaved caspase-3, Bax, and p53. At the same dose, JZ534 showed better antitumor activity than curcumin. These results suggest that JZ534 exhibits excellent anti-lung cancer activity by inhibiting the growth and inducing the apoptosis of lung cancer cells. Therefore, JZ534 is a promising lead compound for cancer treatment.

## 1. Introduction

Lung cancer is a dangerous tumor that threatens human health and life with high morbidity and mortality [1]. The incidence and mortality of lung cancer have significantly increased worldwide [1]. Chemotherapy is the primary treatment for lung cancer and is applied in most cases because this disease is usually diagnosed at an advanced stage [2–5]. However, the efficiency of traditional chemotherapeutic drugs such as cisplatinum is poor. The five-year overall survival of patients treated with traditional chemotherapeutic drugs is not ideal because of adverse effects and drug resistance [6]. Therefore, substantive research has focused on searching for new and effective chemical drugs. Natural medicine is still the main source of new anticancer medicines because of its advantages, such as wide variety of sources and low toxicity.

**Figure 1:**
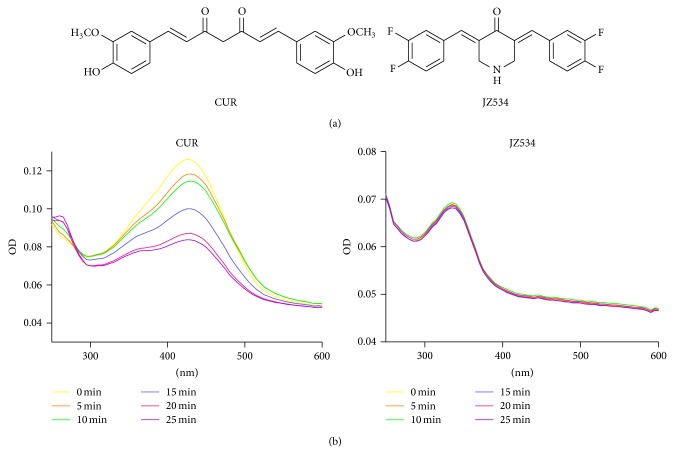
Chemical structures and stability experiment. (a) Structures of JZ534 and curcumin (CUR). (b) Stability tests of JZ534 and curcumin.

Curcumin, a natural polyphenol derived from turmeric (*Curcuma longa*), exerts excellent anticancer activity in vitro [7–10]. Curcumin can restrain cancer cell proliferation and induce cancer cell apoptosis [11–13], such as in head and neck squamous cell cancer [14], breast cancer [15, 16], prostate cancer [17], lung cancer, and pancreatic adenocarcinoma [18]. However, curcumin can be easily catalytically decomposed in vitro or in vivo and only has a low bioavailability in vivo because of the structure of *β*-diketone [19–21]. Therefore, many researchers have modified the structure of curcumin to obtain stable analogs with strong antitumor activities. In our previous work, we designed various monocarbonyl analogs of curcumin (MACs) with no unstable *β*-diketone group. Experiments on degradability in vitro and pharmacokinetics in vivo demonstrated that MACs possess good stability and pharmacokinetic properties [22]. In this study, we identified a new MAC, JZ534 (Figure 1(a)), from compound banks containing 143 MACs. This analog exerts better anti-lung cancer activity than curcumin. Results show that JZ534 can potently inhibit lung cancer cell proliferation and induce cancer cell apoptosis.

## 2. Methods and Materials

### 2.1. Cell Lines, Compounds, and Reagents

The human lung carcinoma cell line H460 was purchased from ATCC (Manassas, VA). The human normal liver cell line HL7702 and the human lung carcinoma cell lines A549, HCC827, and H1975 were purchased from the Cell Library Committee of the Chinese Academy of Sciences (Shanghai, China). All cell lines were grown as monolayers in RPMI-1640 (CA, USA) supplemented with 10% heat-inactivated fetal bovine serum (FBS; Gibco) and 1% penicillin-streptomycin solution (Gibco) at 37°C with 5% CO_2_/95% air. The curcumin analog JZ534 was designed and synthesized in our laboratory. Before the experiments, the curcumin analog JZ534 was purified to >95% by recrystallization and silica gel chromatography. The structure of JZ534 is shown in Figure 1(a). The compound used in vitro was dissolved in DMSO. Anti-cyclin B1, anti-p53, anti-Cdc2, anti-GAPDH, anti-cleaved caspase-3, goat anti-rabbit IgG-HRP, and mouse anti-goat IgG-HRP were purchased from Santa Cruz Biotechnology (Santa Cruz, CA, USA). DMSO was purchased from Sigma (St. Louis, MO). MTT and DMSO were obtained from Sigma-Aldrich (St. Louis, MO, USA). FBS and 0.25% trypsin (containing EDTA) were from HyClone (Logan, UT, USA). Protease phosphatase inhibitor mixture was obtained from Applygen (Beijing, China). Acrylamide (40%), coomassie brilliant blue, TEMED, tris, glycine, sodium dodecyl sulfate (SDS), prestained protein marker, and nonfat dry milk were from Bio-Rad (Germany). PI/RNase staining buffer was purchased from BD Bioscience (Franklin Lakes, NJ).

### 2.2. Stability Test

JZ534 or curcumin (10 *μ*L each) was added into each cuvette. The absorbance was immediately measured from 200 nm to 600 nm to determine the OD of JZ534 and curcumin. OD was detected once every 5 min. Three independent experiments were conducted.

### 2.3. Cell Viability Assays

H460, A549, HCC827, and H1975 cells were seeded onto 96-well plates with 5000 cells per well in 10% heat-inactivated FBS containing RPMI-1640 for 24 h and then treated with different doses of JZ534 and curcumin for 72 h. DMSO served as the negative control. After treatment, cell viability was measured by the MTT assay. Each treatment was conducted in triplicate.

### 2.4. Apoptosis Assay

H460, A549, HCC827, and H1975 cells were grown for 24 h in a six-well plate and then treated with JZ534 or curcumin for 24 h. Then, the cells were digested by trypsin, washed twice with PBS, and suspended in 1x loading buffer to achieve a concentration of 1 × 10^6^ cells per milliliter. The cells were stained with 5 *μ*L of Annexin-V and 1 *μ*L of PI for 15 min at 37°C. The number of cells was determined using flow cytometry. Flow cytometric analysis was performed using a fluorescence-activated cell sorter.

### 2.5. Cell Cycle Analysis

To determine the effects of JZ534 on the cell cycle, A549, H460, H1975, and HCC827 were cultivated for 24 h in RPMI1640 without FBS and then treated with 2 *μ*M JZ534 or curcumin for 24 h. The cells were digested by trypsin, collected, and then washed twice with ice-cold PBS. The percentages of cells in the G_0_/G_1_, S, and G_2_/M phases were determined using flow cytometry.

### 2.6. Colony Formation Assay

A549 and H460 cells were plated in six-well plates and cultured overnight. JZ534 or curcumin at 4 *μ*M was incubated with A549 and H460 cells for 24 h and then replaced with fresh culture fostered for 1 week. The number of surviving colonies was counted after staining with crystal violet solution and then photographed. DMSO served as the negative control, and the experiment was conducted at least in triplicate.

### 2.7. Western Blot Assay

The Western blot assay was used to evaluate the preliminary mechanism. Cell lysates were added at 60 *μ*L/well into a six-well plate. The cells were lysed, and debris was removed by centrifugation at 12,000 rpm for 10 min at 4°C. The supernatant was run on a 10% SDS-PAGE gel at 120 V and then transferred onto a polyvinylidene difluoride membrane. After being blocked with 5% nonfat dry milk in TBST for 1.5 h, the membrane was incubated with the primary antibody (anti-cyclin B1, anti-p53, anti-CDC, anti-GAPDH, and anti-cleaved caspase-3) overnight and then incubated with goat anti-rabbit IgG and HRP-linked antibody for 1 h. The blots were detected with an ECL detection kit according to the manufacturer's procedure. The results were analyzed by Quantity One software to determine the relative band density ratio. GAPDH was used as the standard, and the ratio between the analyzed protein and GAPDH from quantitative densitometric analysis was used to compare the control and experimental samples.

### 2.8. Statistical Analysis

All experiments were repeated in triplicate (*n* = 3). The data were expressed as means ± SEM. All statistical analyses were performed using GraphPad Prism 5.0 (GraphPad, San Diego, CA). Student's *t*-test and two-way ANOVA were used to analyze the differences between the sets of data. Statistical significance was considered at *P* < 0.05.

## 3. Results

### 3.1. Stability Assay of JZ534

The stability of JZ534 was analyzed in phosphate buffer (pH 7.4) by an absorption spectrum assay. The optical density (OD) of curcumin displayed maximum intensity at 425 nm, and the absorption peak intensity decreased in a time-dependent manner (Figure 1(b)). However, the absorption peak of JZ534 did not decrease at the maximum absorption wavelength with time (Figure 1(b)). The result of the stability assay suggested that JZ534 was more stable than curcumin.

### 3.2. JZ534 Inhibited Lung Cancer Cell Proliferation

Lung cancer cell lines (H460, A549, H1975, and HCC827) were employed in this study, and the methylthiazoletetrazolium (MTT) assay was performed to measure the inhibitory effect of JZ534 on tumor cell proliferation. JZ534 demonstrated satisfactory inhibitory activities against the four lung cancer cell lines, with 50% inhibiting concentration values (IC_50_) of <5 *μ*M. The IC_50_ values of curcumin against these four lung cancer cell lines were all greater than 17 *μ*M. Therefore, JZ534 exhibited better anti-lung cancer activity than curcumin.

**Figure 2:**
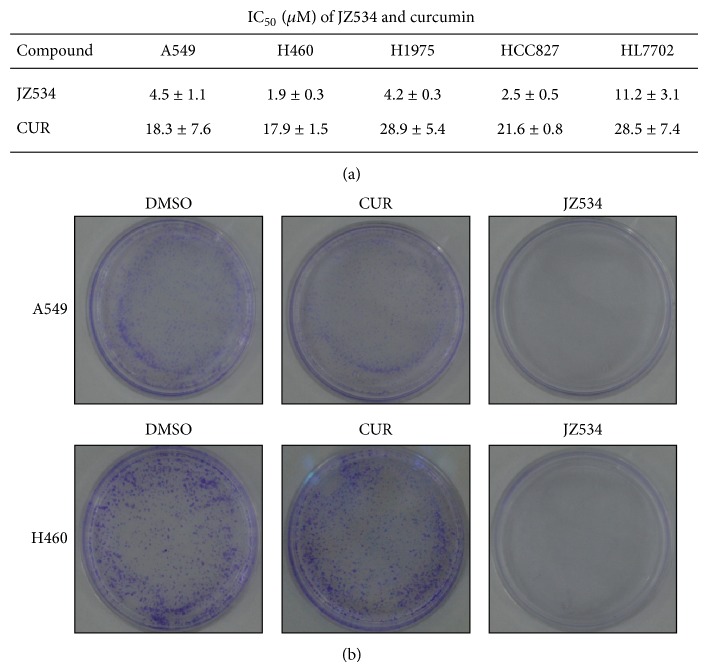
JZ534 inhibited lung cancer cell proliferation. (a) H460, A549, H1975, HCC827, and HL7702 were incubated with JZ534 (0.5, 1, 2, 4, and 8 *μ*M) or curcumin (0.096, 0.48, 2.4, 12, and 60 *μ*M) for 72 h; OD values were tested by MTT, and then the inhibition rate and IC_50_ were calculated. The assay was performed in triplicate. Data are presented as the means ± SD. (b) The number of colonies was counted after A549 and H460 cells were incubated with 4 *μ*M JZ534 or curcumin for a week and stained with crystal violet.

The antiproliferation effects of JZ534 on H460 and A549 were further investigated using colony-forming experiments. After A549 and H460 cells were incubated with JZ534 or curcumin (4 *μ*M) for 1 week, cell colonies were stained with crystal violet and then counted. Compared with dimethyl sulfoxide (DMSO), JZ534 obviously suppressed the colony formation of lung cancer cells, whereas curcumin showed no significant inhibitory effect on cell colony formation (Figure 2(b)). This finding is consistent with the results of the MTT assay.

### 3.3. JZ534 Induced G_2_/M Cell Cycle Arrest in Lung Cancer Cells

**Figure 3:**
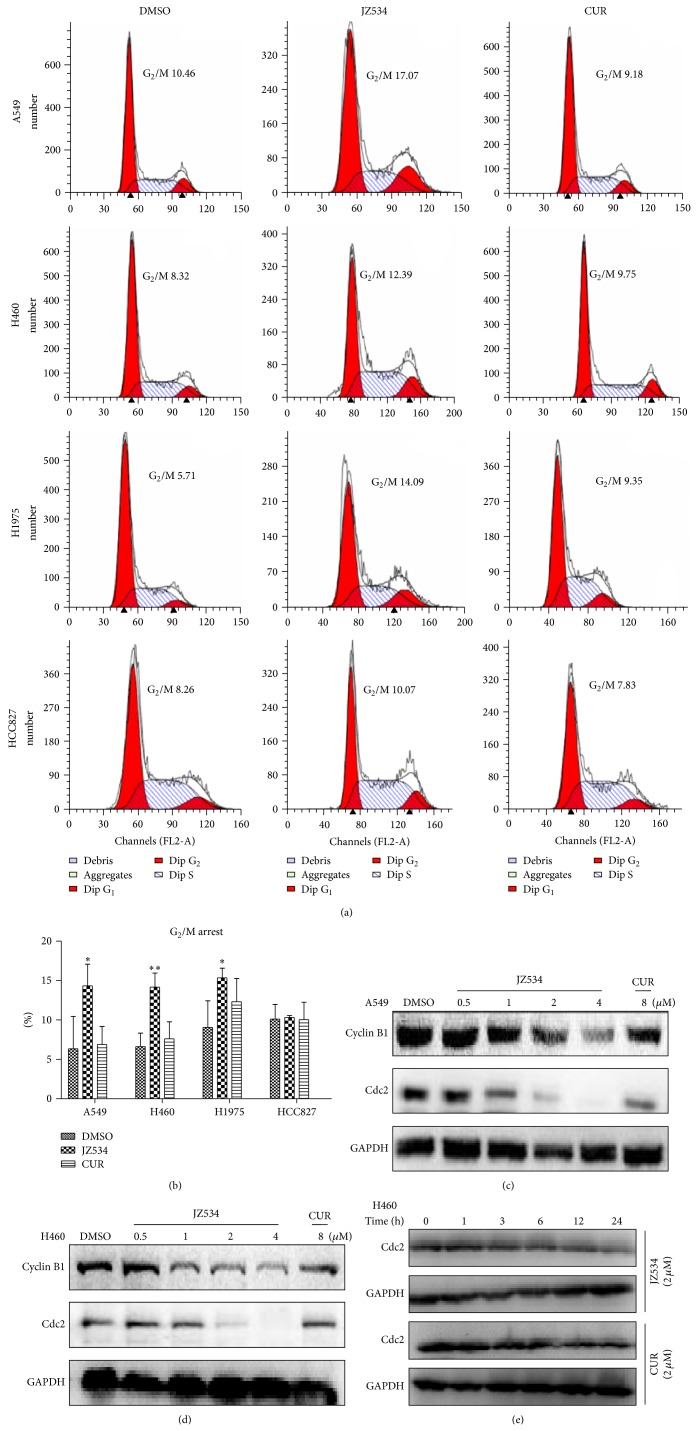
JZ534 induced lung cancer cell cycle arrest. (a) H460, A549, H1975, and HCC827 were grown for 24 h in RPMI1640 without FBS and then treated with 2 *μ*M JZ534 or curcumin for 24 h. The numbers of cells in the G_0_/G_1_, S, and G_2_/M phases were determined by flow cytometry. (b) Histogram illustrating the rate of cells in the G_2_/M phase from three flow cytometric analyses from three separate treatments. The mean of three values was determined (^∗^ *P* < 0.05; ^∗∗^ *P* < 0.01). ((c), (d), (e)) Cell cycle-related proteins cyclin B1 and Cdc2 were detected by Western blot analysis. (c) A549 cells and (d) H460 cells were incubated with different concentrations of JZ534 or curcumin for 24 h. (e) H460 cells were incubated with 2 *μ*M JZ534 or curcumin for different times.

Flow cytometry was performed to determine whether or not the inhibitory effect of JZ534 on lung cancer cell growth was through cell cycle arrest. A549, H460, H1975, and HCC827 were treated with 2 *μ*M JZ534 or curcumin. The numbers of cells in the G_0_/G_1_, S, and G_2_/M phases were counted by flow cytometry. As shown in Figures 3(a) and 3(b), JZ534 arrested all the cancer cell lines, except for HCC827, in the G_2_/M phase. Meanwhile, curcumin at the same concentration exerted no effect on the cell numbers of the four cells in the G_2_/M phase.

### 3.4. JZ534 Inhibited the Expression of Cell Cycle-Related Proteins in Lung Cancer Cells

To explore the mechanism of cell cycle arrest, the cell cycle-related proteins Cdc2 and cyclin B1 were examined by Western blot. After treatment with 2 *μ*M JZ534, Cdc2 time-dependently decreased in H460 cells (Figure 3(e)). After A549 and H460 were treated with JZ534 at various concentrations (0.5, 1.0, 2.0, and 4.0 *μ*M) or with curcumin at 8 *μ*M for 24 h, the expression levels of cyclin B1 and Cdc2 were reduced in a dose-dependent manner (Figures 3(c) and 3(d)). Meanwhile, curcumin exerted no significant effect on the expression levels of cyclin B1 and Cdc2. Thus, JZ534 arrested the cells in the G_2_/M phase by reducing the expression of cell cycle-related proteins.

### 3.5. JZ534 Induced Apoptosis in Lung Cancer Cells

**Figure 4:**
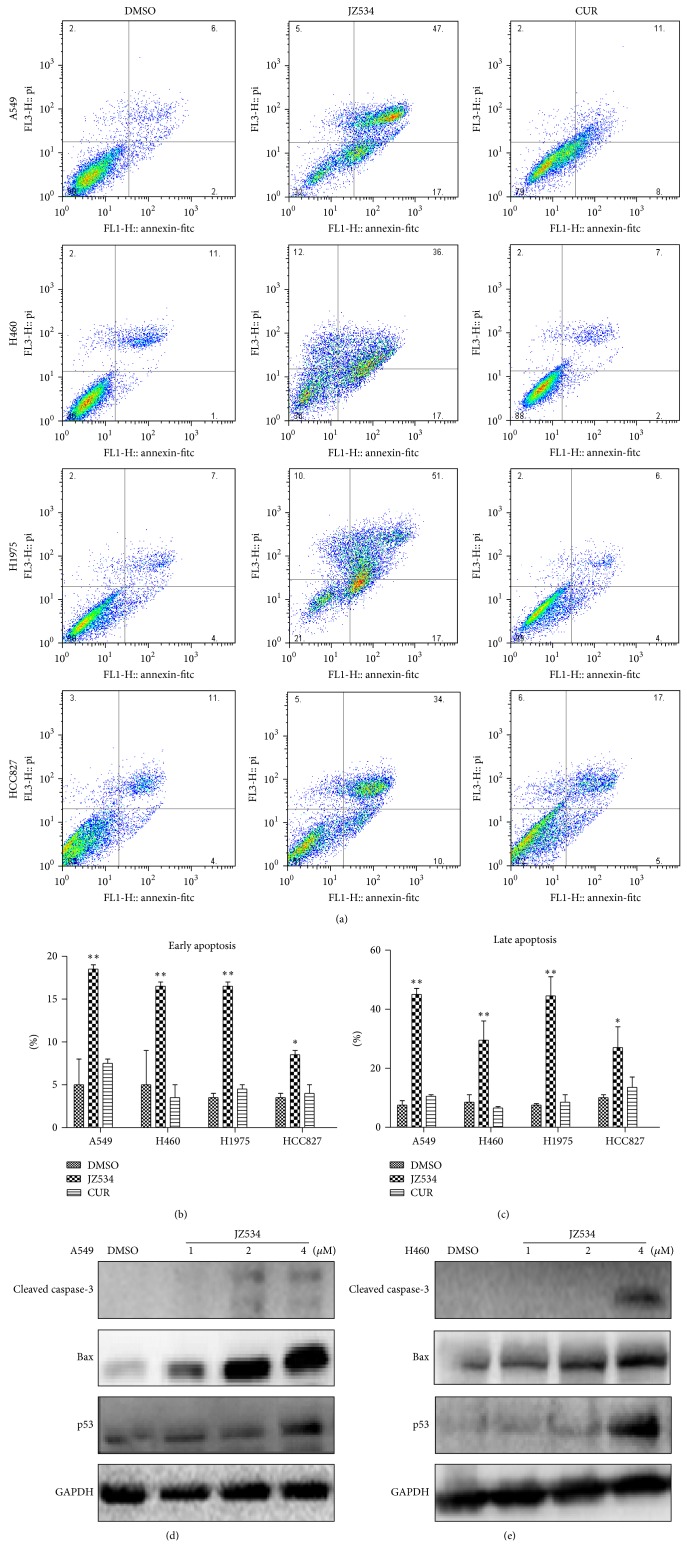
JZ534 induced the cell apoptosis of lung cancer cells. (a) H460, A549, H1975, and HCC827 were incubated with 8 *μ*M JZ534 or curcumin for 24 h. Percentage of apoptotic cells as determined by flow cytometry. ((b), (c)) Histogram illustrating the rate of cell apoptosis from three FACS analyses from three separate treatments (^∗^ *P* < 0.05; ^∗∗^ *P* < 0.01). ((d), (e)) Cell apoptosis was detected by Western blot analysis. Cleaved caspase-3, Bax, and p53 were detected in H460 and A549 cells incubated with different concentrations of JZ534 or curcumin for 24 h.

To further study the internal mechanism of cell proliferation inhibition observed from the MTT assay, we examined the apoptotic effects of JZ534 on the lung cancer cells via flow cytometry. A549, H460, H1975, and HCC827 cells were treated with 8 *μ*M JZ534 or curcumin for 24 h, and then early- and late-stage apoptosis were studied by using Annexin-V/propidium iodide (PI) staining. Compared with DMSO, JZ534 obviously increased the early- and late-stage apoptosis rates of the four lung cancer cells; the late-stage apoptosis rates of the cells were all greater than the early-stage apoptosis rates (Figures 4(a), 4(b), and 4(c)).

### 3.6. JZ534 Induced the Expression of Apoptosis-Related Proteins in Lung Cancer Cells

To elucidate the mechanism of JZ534-inducing apoptosis, the expression levels of apoptosis-related proteins—cleaved caspase-3, Bax, and p53—were examined by Western blot in A549 and H460 cells. Results showed that 2 *μ*M JZ534 activated the expression levels of cleaved caspase-3 and Bax in A549 cells and that 4 *μ*M JZ534 activated p53 in A549 cells (Figure 4(d)). The expression levels of cleaved caspase-3, Bax, and p53 significantly increased in the H460 cells incubated with 4 *μ*M JZ534 (Figure 4(e)). The results indicated that JZ534 induced lung cancer cell apoptosis by activating apoptosis-related proteins.

## 4. Discussion

Chemotherapeutic agents based on natural medicine are a common source of oncotherapeutic drugs. Curcumin analogs have attracted considerable attention because of their antitumor activities. Compared with other curcumin analogs, MACs demonstrate better metabolic stability and possess better pharmacological activity. In our previous research, we designed and synthesized aliphatic ketone MACs, whose 5-carbon linker contains aliphatic ketones, including 5-diaryl-1,4-pentadiene-3-ones, together with cyclopentanone and cyclohexanone. We screened several analogs with satisfactory antitumor activities, such as B19 [23, 24] and B63 [25]. Then, we designed and synthesized heterocycle ketone MACs, whose 5-carbon linker is a piperidin-4-one structural unit, and found multiple analogs with satisfactory antitumor activities. In the present study, we investigated the anticancer effect of the curcumin analog JZ534 on lung cancer cell lines. Compared with curcumin, JZ534 not only displayed higher stability but also exhibited better antitumor activity.

Lung cancer has the highest mortality rate among all cancer types worldwide. Chemotherapy is the main therapeutic method for lung cancer and the development of novel therapeutic agents is especially urgent because patients of this disease always have a poor prognosis and are diagnosed at an advanced stage [1–5].

In the current study, we revealed that the novel compound JZ534 can be developed into a therapeutic agent against lung cancer. JZ534 potently inhibited the proliferation of lung cancer cells and induced cell cycle arrest and apoptosis. The IC_50_ values of JZ534 on H460, A549, H1975, and HCC827 were all under 5 *μ*M, whereas those of curcumin were all above 10 *μ*M. Fewer doses of JZ534 can induce apoptosis and cell cycle arrest, however curcumin exerted no significant effect on lung cancer cells at the same dose. Thus, JZ534 displayed better antitumor activity than curcumin.

Curcumin can inhibit tumor growth by regulating the expression of cycle-related genes and proteins [26]. Cyclin B1 and Cdc2 are keys to G_2_/M cell cycle checkpoints. In the present study, JZ534 induced G_2_/M phase cell cycle arrest in H460 and A549. The expression of the G_2_/M-related proteins cyclin B1 and Cdc2 decreased in a dose-dependent manner after JZ534 treatment. The effect of JZ534 was better than that of curcumin at the same dose.

Proteins such as cleaved caspase-3, Bax, and p53 are crucial in cell apoptosis [27, 28]. Many anticancer drugs induce cancer cell apoptosis by upregulating the expression levels of cleaved caspase, Bax, and p53 [29]. Curcumin can activate the signaling pathway of p53, and increase the expression of p53, and enhance the binding activity of p53 DNA, which upregulates the expression of the p53, downstream effector Bax and thus induces tumor cell apoptosis [30]. The curcumin analog B19 displays anti-lung cancer activity by inducing the expression of cleaved caspase-3 and p53 [31]. In the present research, high concentrations of JZ534 activated caspase-3 in lung cells. In addition, the expression levels of p53 and Bax increased in a dose-dependent manner. This result suggested that JZ534 may induce apoptosis by activating apoptosis-related proteins.

## 5. Conclusion

In summary, our results reported a new MAC, namely, JZ534, which exhibited excellent anti-lung cancer activity. JZ534 can significantly inhibit the proliferation and the colony formation of lung cancer cells, induce cell cycle arrest by downregulating the cycle-related proteins cyclin B1 and Cdc2, and significantly induce cell apoptosis by upregulating the apoptosis-related proteins cleaved caspase-3, Bax, and p53. Meanwhile, JZ534 displayed higher stability and better antitumor activity than curcumin. Therefore, JZ534 is a promising lead compound in the treatment of lung cancer. However, the exact mechanism of JZ534 needs further analysis.
